# Mild hypothermia during cardiopulmonary bypass assisted CABG is associated with improved short- and long-term survival, a 18-year cohort study

**DOI:** 10.1371/journal.pone.0273370

**Published:** 2022-08-25

**Authors:** K. D. W. Hendriks, J. N. Castela Forte, W. F. Kok, H. E. Mungroop, H. R. Bouma, T. W. L. Scheeren, M. Mariani, R. H. Henning, A. H. Epema

**Affiliations:** 1 Department of Clinical Pharmacy and Pharmacology, University Medical Centre Groningen, University of Groningen, Groningen, The Netherlands; 2 Department of Anaesthesiology, University Medical Centre Groningen, University of Groningen, Groningen, The Netherlands; 3 Department of Internal Medicine, University Medical Centre Groningen, University of Groningen, Groningen, The Netherlands; 4 Department of Cardiothoracic Surgery, University Medical Centre Groningen, University of Groningen, Groningen, The Netherlands; Case Western Reserve University School of Medicine, UNITED STATES

## Abstract

Data substantiating the optimal patient body temperature during cooling procedures in cardiac operations are currently unavailable. To explore the optimal temperature strategy, we examined the association between temperature management and survival among patients during cardiopulmonary bypass assisted coronary artery bypass grafting (CABG) procedures on 30-days and 5-year postoperative survival. Adult patients (n = 5,672, 23.6% female and mean (SD) age of 66 (10) years) operated between 1997 and 2015 were included, with continuous measured intraoperative nasopharyngeal temperatures. The association between mortality and patient characteristics, laboratory parameters, the lowest intraoperative plateau temperature and intraoperative cooling/rewarming rates were examined by multivariate Cox regression analysis. Machine learning-based cluster analysis was used to identify patient subgroups based on pre-cooling parameters and explore whether specific subgroups benefitted from a particular temperature management. Mild hypothermia (32–35°C) was independently associated with improved 30-days and 5-year survival compared to patients in other temperature categories regardless of operation year. 30 days and 5-year survival were 98% and 88% in the mild hypothermia group, whereas it amounted 93% and 80% in the severe hypothermia (<30°C). Normothermia (35–37°C) showed the lowest survival after 30 days and 5 years amounting 93% and 72%, respectively. Cluster analysis identified 8 distinct patient subgroups principally defined by gender, age, kidney function and weight. The full cohort and all patient subgroups displayed the highest survival at a temperature of 32°C. Given these associations, further prospective randomized controlled trials are needed to ascertain optimal patient temperatures during CPB.

## Introduction

Lowering of body temperature has been used for almost seven decades in different cardiac operations, such as during cardiopulmonary bypass (CPB) assisted coronary artery bypass grafting (CABG). Lowering temperature decreases metabolism and therefore limits oxygen requirements directly, and acts via several neuronal and cellular pathways, exerting protective effects [1]. However, low body temperature may also exert negative effects and is therefore considered a potential risk factor. For example, hypothermia induces coagulopathy, with a lowering of a single degree increasing the risk of blood loss by 16% [2], and disrupts cellular anti-oxidant defence [3]. Similarly, low arterial CPB perfusion temperature is associated with increased post-operative acute kidney injury (AKI) [4].

Given the hybrid effects of hypothermia, the optimal temperature management during CPB is still debated [5]. However, while data from daily practice show a significant range in body temperatures during CPB assisted cardiac surgery [6], there is a paucity of data on hard endpoints, i.e. mortality. While the main focus of temperature management research addresses the target temperature during CPB, other temperature related factors may also affect postoperative morbidity and mortality. For instance, studies in CPB and resuscitation suggest an impact of cooling and/or rewarming rate and of excess rewarming on morbidity and mortality [7–9].

One way of exploring whether there is an optimal body temperature during CPB is analysis of prospectively collected cohort datasets. Such approach has been shown to closely match outcomes of randomized, controlled studies [10, 11]. Therefore, we analysed the association between intraoperative nasopharyngeal temperature during CPB and mortality, by studying routinely collected perioperative data, intraoperative temperature characteristics during CPB assisted CABG and 30-days and 5-year survival in a longitudinal prospectively collected cohort study spanning 18 years [12]. Characteristics of temperature included the lowest plateau nasopharyngeal temperature during CPB and the rates of cooling and rewarming. Given the long study period, the dataset was subdivided in three consecutive time periods for analysis and compared to the entire dataset. In addition, we performed machine learning-based clustering analysis on routinely collected preoperative data to identify patient subgroups and explore whether specific subgroups benefitted from a particular temperature management.

## Materials and methods

### Study design and patient selection

This cohort study was performed according to the STROBE guidelines. The study was conducted in the University Medical Centre Groningen, The Netherlands, and approved by the local Ethics Committee (METC #2010/118) by waiving informed consent.

Data of all adult patients (18 years or older) who underwent elective CABG surgery (patients with additional valve surgeries were excluded) with CPB between 1997 and 2015 in our tertiary care centre were obtained from the cardiothoracic operation registry (CAROLA) and the hospital database (n = 5,815 adult patients). Survival data were obtained from the Dutch Municipal Database (a nation-wide actual registry of birth and death). Patients with a lowest nasopharyngeal temperature < 20°C (n = 8) or with absence of nasopharyngeal temperature data due to the interruption of data output of the CPB machine software to the database (n = 135) were excluded from further analyses. In total 5,672 patients were included. Patient characteristics, perioperative data from 7 days before until 7 days after surgery and survival were analysed.

Survival was examined at 30 days and 5 years of follow-up as commonly used for short and long-term survival in cardiac patient [13]. Moreover, the 5 years endpoint enabled us to include relative recent operations, reflected in a 5-year follow-up of 90.6%.

### Patient characteristics

To maximize completeness and reliability of the dataset, we restricted included variables to those available during the entire 18 years study period and automatically registered by CAROLA and the hospital database. Patient characteristics included age, sex, body mass index (BMI) and EuroSCORE. Pre-operative factors included were haemoglobin, blood glucose, C-reactive protein, platelet and leukocyte counts, urea and creatinine levels. Perioperative characteristics were CPB duration and nasopharyngeal and CPB temperature. Post-operative we looked to the occurrence of acute kidney injury (AKI) and survival.

### CPB temperature protocol

A standardized perfusion protocol was used in all patients, consisting of a CPB index flow of 2.4 L min^-1^m^-2^ with α-stat pH management based on standardized blood gas analysis, mean arterial blood pressure between 60–90 mmHg using phenylephrine or nitro-glycerine as needed [12]. Target temperatures during CPB were left at the discretion of the operative team. Perioperative nasopharyngeal temperature (Tnp) was continuously measured and used to define the lowest plateau body temperature and to calculate cooling and rewarming rates.

### Body temperature categorization

Categorical temperature groups were defined based on the lowest Tnp during CPB lasting longer than 5 min. Temperature categories consisted of normothermia (35.1–37.5°C), mild (32.1–35°C), moderate (30–32°C) and severe hypothermia (<30°C). In addition, data were analysed per °C.

### Calculation of cooling and rewarming rate

Cooling rate was calculated from the continuous temperature logs, starting by the timepoint when Tnp dropped with ≥ 0.2°C/min until the decrease fell below 0.1°C/min. Similarly, rewarming rate was calculated with the reverse algorithm.

### Clustering analysis

Clustering analysis is a form of unsupervised machine learning, where unlabelled data is split into different sub-groups (clusters) based on inherent similarities between data points [14]. It has been increasingly used in exploratory data analyses for identifying relevant patient sub-groups without any a priori hypotheses [15]. We used k-means clustering to study whether routinely collected pre-cooling parameters could identify relevant patient sub-groups with different survival for distinct intraoperative temperature strategies. K-means divides data into a user-defined number of clusters, which is repeatedly updated until the distance (i.e. difference) between points within a cluster is minimized [16]. Eight clinical features including patient characteristics (age, gender, and body mass index) and preoperative laboratory values (urea, creatine, haemoglobin, thrombocytes and leukocytes) were used as input variables. The optimal number of clusters was selected based on the calculated silhouette score, visual representation of the clusters using principal component analysis (S1 Fig), and optimal differentiation of input parameters. Significance of the differences in input features and outcome variables between clusters was determined using analysis of variance and χ^2^ test.

### Statistical analysis

Normality of data distribution was tested by the Kolmogorov-Smirnov test. Differences in baseline characteristics were examined using a Mann-Whitney test in case of continuous data and Pearson’s chi-square for nominal data using two-tailed tests. Survival was analysed at 30 days and 5 years after the operation, thus representing the highest number of patients with full follow-up in all temperature groups. Five years mortality was corrected for 30 days mortality. Kaplan Meier curves and Log-Rank χ^2^ tests were used to analyse postoperative survival. Multivariable Cox regression with calculations of hazard ratio (HR) and odds ratio (OR) was used to assess mortality risk.

### Software and data presentation

Statistical calculations were performed using SPSS 22.0 for Windows. Clustering analysis was conducted using R version 3.6.1. Figures were made using Graphpad Prism 6.0 and the ggplot2 package in R. Data are presented as absolute numbers with percentages, mean ± SD, or median (25th-75th percentile). Differences were considered significant at p<0.05.

## Results

### Patient characteristics

Patients had a mean (SD) age of 66.0 (9.7) and had a BMI of 27.3 (3.8) kg/m^2^. The majority of procedures were performed in male patients (76%). During CPB, the lowest plateau body temperature ranged from 20 to 37°C, with 85% of operations between 30 and 35°C. Further characteristics, including EuroSCORE, biochemistry analysis and perfusion details are outlined in Table 1.

**Table 1:** Patient characteristics, pre- and perioperative parameters.

	total	Normothermia	Mild	Moderate	Severe	p-value
		35–37°C	32–35°C	30–32°C	<30°C	
**Patient characteristics**	(n = 5672)	261 (4.6%)	1947 (34.3%)	2899 (51.1%)	566 (10.0%)	
Age (years)	66 ± 9.7	65 ± 9.5	66 ± 9.7	66 ± 9.6	66 ± 9.9	n.s
Female gender	24%	28%	22%	23%	28%	0.014
BMI (kg∙m^-2^)	27.3 ± 3.5	27.8 (25–30)	27 (25–30)	27 (25–29)	27.1 (24–29)	<0.01
Weight	82.1 ± 13.1	83 ± 13.6	83 (74–92)	81 (72–90)	80 (72–89)	<0.01
EuroSCORE						0.002
Low (<2%)	768 (24.4)	41 (19.6)	351 (25.7)	339 (23.9)	37 (23.7)	
Medium (2–5%)	1044 (32.2)	51 (24.4)	453 (33.2)	499 (35.1)	41 (26.3)	
High (>5%)	1337 (42.5)	117 (56)	560 (41.1)	682 (41)	78 (50.0)	
**Preoperative blood and serum samples**						
Haemoglobin (mmol/l)	8.1 (7.5–9.1)	8.2 (7.6–9.1)	8.3 (7.7–9.1)	8.1 (7.4–9.0)	7.7 (6.6–8.9)	<0.01
Platelet count (x10^9/l)	237 (188–276)	241 (190–275)	242 (191–280)	236 (188–275)	223 (166–269)	<0.01
Leukocyte count (x10^3/l)	8.2 (6.4–9.2)	8.7 (7.0–9.9)	8.3 (6.5–9.5)	8.0 (6.3–9.0)	8.2 (6.5–9.5)	<0.01
C-reactive protein (mg/l)	24.7 (7–30)	27.3 (25–30)	22 (6–26)	25 (6–33)	32 (9–37)	0.193
Glucose (mmol/l)	7.2 (5.3–8.6)	6.8 (5.5–7.1)	7.1 (5.3–8.2)	7.1 (5.3–8.6)	7.66 (5.3–9.3)	0.050
eGFR	78 (60–93)	93 (65–107)	82 (62–99)	75 (58–90)	73 (56–87)	<0.01
**Perioperative parameters**						
Duration of perfusion (min)	102 (75–118)	95 (69–116)	95 (70–112)	104 (79–120)	116 (86–130)	<0.01
Aortic cross clamp time (min)	58 (43–73)	35 (0–60)	53 (38–66)	62 (47–76)	67 (49–80)	<0.01
**Post-operative (6h) parameters**						
Haemoglobin (mmol/l)	5.6 (5.2–6.1)	5.8 (5.3–6.4)	5.7 (5.2–6.2)	5.6 (5.1–6.0)	5.6 (5.1–6.1)	<0.01

Data presented as mean ± SD, number (proportion) or median (25^th^– 75^th^ percentile). EuroSCORE gives the 30 days mortality risk. Significance tested by oneway ANOVA.

For analyses, we categorized the lowest nasopharyngeal temperature during CPB into 4 groups, based on the nasopharyngeal temperature (Tnp): normothermia (35–37.5°C, n = 261), mild hypothermia (32–35°C, n = 1947), moderate hypothermia (30–32°C, n = 2899) and severe hypothermia (<30°C, n = 566). Of note, only 15% of the cohort was operated with a Tnp below 30°C or above 35°C. With the exception of a relative lower EuroScore in the lowest and highest temperature groups (Table 1), no clinically relevant differences were observed between temperature categories.

### CPB temperature and postoperative survival

**Fig 1:**
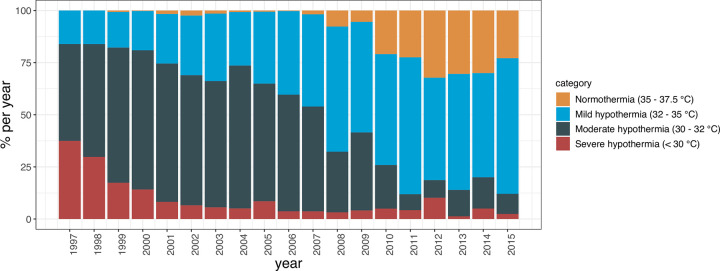
Trend in nasopharyngeal temperatures during CPB assisted CABG over time.

**Fig 2:**
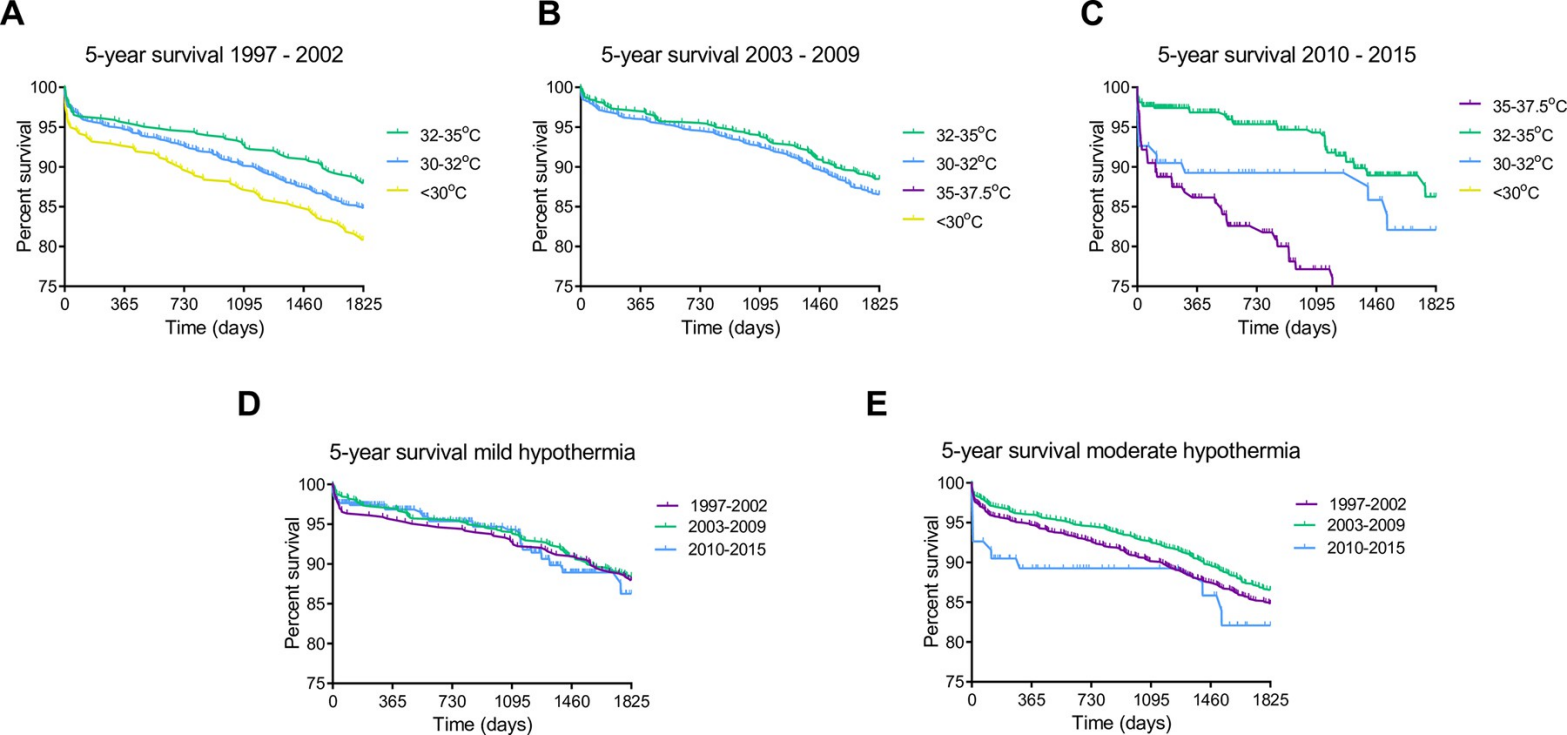
5 year survival per categorized minimal nasopharyngeal temperature per time period, including (a) 1997–2002, (b) 2003–2009 and (c) 2010–2015.

Temperature management during CPB was left at the discretion of the operative team. As their preferences regarding targeted Tnp and details of procedures may have changed over time, we first examined the distribution of temperature categories per year (Fig 1). Throughout the years, a clear shift from lower to higher Tnp during CPB occurred. For instance, from 1997 to 2002, severe and moderate hypothermia were regularly chosen as temperature strategy resulting in the majority of operations being performed at Tnp below 32°C. Contrary, in the most recent period (2010–2015), severe hypothermia had been virtually abandoned, yet normothermia became increasingly popular with most of the patients being operated at Tnp above 32°C. Given the differences in temperature management over time, we further analysed survival by splitting the cohort in three time periods with minimal variation in the percentage of patients with the lowest and highest temperature categories. Kaplan-Meier plots were generated for each time period, in which patient survival of different temperature categories were depicted when they represented at least 10% of total operations during that time period. Irrespective of the time periods analysed, mild hypothermia was associated with the highest 5-years survival rates, respectively 88%, 88% and 85% throughout the three time frames (Fig 2A–2C). Further, patients categorized as severe hypothermia (in 1997–2002) and normothermia (2010–2015) showed with 79% and respectively 66% the lowest survival rates. In agreement, overall survival of the full cohort was stable over time, despite possible changes in surgical techniques and materials (Fig 2D, 2E).

**Fig 3:**
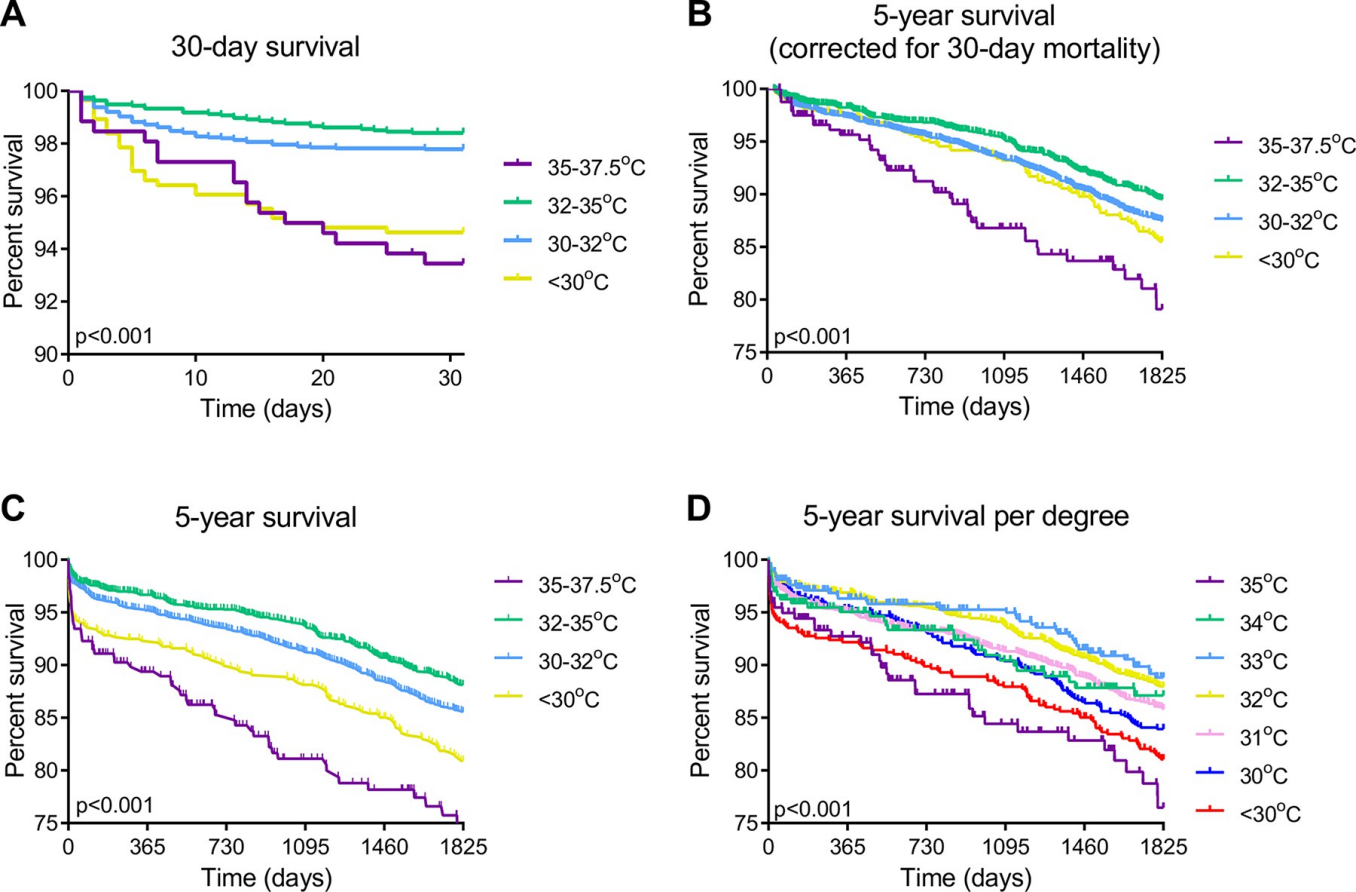
Survival per minimal nasopharyngeal temperature. (a) 30-day survival, (b) 5-year survival corrected for 30-day survival (31 days– 5 year), (c) 5-year survival and (d) 5-year survival per degree.

Next, we analysed survival in the full cohort in more detail. Expectedly, given the time period analysis, Tnp during CPB was significantly associated with survival both in the 30-day and 5-year follow-up (Fig 3A, 3C; Log rank χ^2^ = 54, p <0.001, Log rank χ^2^ = 52, p <0.001, respectively). The relationship between Tnp and 30-day mortality displayed a clear dichotomy. Whereas patients with mild and moderate hypothermia showed high 30-day survival (both 98%), the more extreme groups of severe hypothermia and normothermia showed significantly lower survival, amounting 93% (Fig 3A; Log rank χ^2^ = 54, p <0.001). Notably, after correction for the 30-day mortality, 5-year follow-up showed a comparable pattern, with mild hypothermia being associated with the highest 5-year survival compared to all other temperature categories (Fig 3B; Log rank χ^2^ = 21, p <0.001). Whereas overall 5-year survival in our cohort was 85%, normothermia was associated with the lowest 5-year survival amounting 72% (Fig 3C). In contrast, mild hypothermia associated with the highest survival (88%, Fig 3C). Other temperature categories showed in-between survival rates (Fig 3C). To further refine this analysis, mild and moderate hypothermia groups were subdivided per °C. At 5 years follow-up, the survival was highest after procedures with a Tnp temperature of 32 and 33°C, which was significantly higher than survival observed with higher and lower temperatures (Fig 3D; Log rank χ^2^ = 62, p <0.001).

### Cooling and rewarming rates

**Fig 4:**
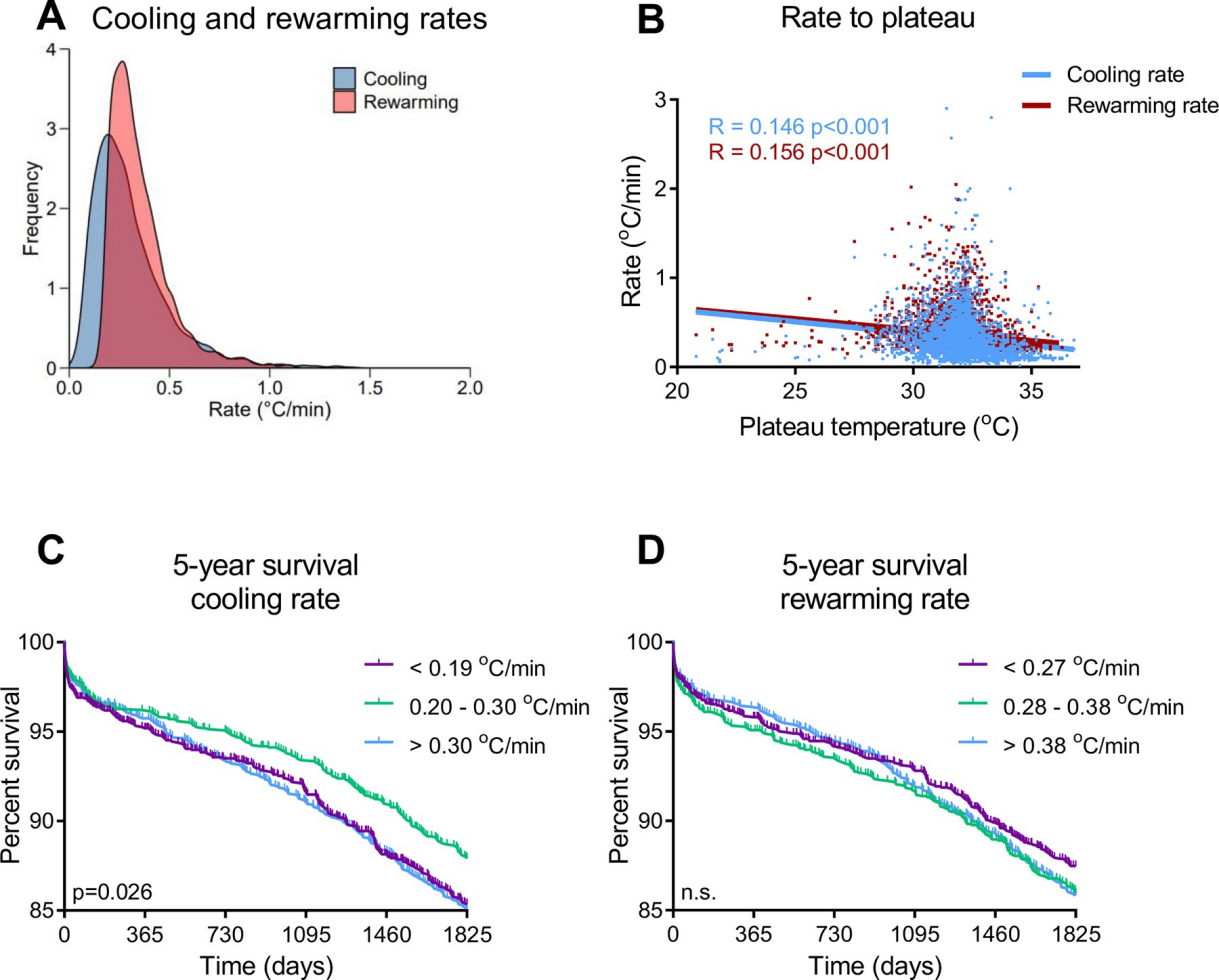
Survival relative to cooling and rewarming rate. (a) Density plot of the cooling and rewarming rates. (b) Scatterplot cooling and rewarming rates with plateau temperature (c) Cooling rate– 5-year survival (d) Rewarming rate– 5-year survival.

During CABG, patient temperature is mainly controlled by the arterial outlet temperature of the CPB machine. We found a large variation in the rate of cooling and rewarming, ranging from 0.02 to 2.8°C/min in our full cohort (Fig 4A). Both rates showed a weak association to plateau Tnp (Fig 4B, cooling R = -0.146 p<0.001, rewarming R = -0.156 p<0.001) and a marginal correlation was found between cooling rate and BMI (R = -0.037 p = 0.013). For further analysis we categorized the rates in tertiles. In Fig 4C, we outlined survival per cooling rate. The middle tertile (cooling rate 0.20–0.30°C/min) associated with the highest survival, with both slower and faster cooling rates displaying lower survival (Log rank χ^2^ = 7, p<0.026, OR intermediate relative to slower cooling 0.771, 95% CI 0.631 to 0.941, p = 0.010). In contrast, no relationship was found between rewarming rates and survival (Fig 4D).

### Temperature characteristics corrected for routinely collected data

To examine whether temperature variables independently predict survival, Cox multivariable regression analysis was performed for both 30-day and 5-year survival based on routinely collected data, including temperature data, patient characteristics, pre- and perioperative parameters and lab chemistry. Cox regression showed that mild and moderate hypothermia independently associate with both short and long-term survival (Table 2).

**Table 2:** Multivariate COX regression analysis.

	30-days survival HR (95% CI)	p-value	5-year survival HR (95% CI)	p-value
**Patient characteristics**				
Age (years)	1.02 (0.99–1.06)	0.179	1.06 (1.04–1.07)	<0.001
Female gender	0.60 (0.31–1.16)	0.130	0.60 (0.50–0.84)	0.001
BMI (kg∙m^-2^)	0.98 (0.91–1.05)	0.509	0.99 (0.97–1.03)	0.808
**Preoperative parameters**				
Haemoglobin (mmol/l)	0.91 (0.72–1.13)	0.386	0.95 (0.87–1.05)	0.304
Platelet count (x10^9/l)	1.00 (0.997–1.00)	0.940	1.00 (1.00–1.00)	0.002
**EuroSCORE**				
Low risk (<2.5%)	Reference		Reference	
Medium risk (2.5–5%)	0.242 (0.06–0.99)	0.048	0.96 (0.66–1.40)	0.826
High risk (>5%)	2.11 (0.81–5.52)	0.126	1.43 (0.98–2.08)	0.061
**Acute kidney injury**	6.28 (3.64–10.87)	<0.001	2.39 (1.88–3.04)	<0.001
**Perioperative parameters**				
Duration of perfusion (15 min)	1.10 (1.03–1.17)	0.003	1.11 (1.07–1.14)	<0.001
**Temperature strategy**				
Normothermia (35–37°C)	Reference		Reference	
Mild hypothermia (32–35°C)	0.15 (0.07–0.34)	<0.001	0.36 (0.25–0.52)	<0.001
Moderate hypothermia (30–32°C)	0.24 (0.12–0.50)	<0.001	0.39 (0.27–0.56)	<0.001
Severe hypothermia (<30°C)	0.61 (0.25–1.53)	0.294	0.52 (0.31–0.87)	0.01

Hazard ratio (HR) with 95% confidence interval (C.I.) on mortality in CABG surgery. EuroSCORE gives the 30 days mortality risk.

### Identification of pre-cooling phenotypes by clustering analysis

To explore whether mild hypothermia is the optimal temperature strategy for all CABG procedures or whether specific patient subgroups may benefit from individualized Tnp during CPB, we identified patient subgroups using clustering analysis. As k-means clustering does not allow for missing data, the 4,345 patients without missing inputs were analysed. The optimal number of clusters was determined to be 8 (S1 Fig). The distribution of cluster membership and between-cluster difference for each input variable can be found in Table 3 and S1 File. Clear differences between clusters were found. Interestingly, clusters 5 and 7 include only female patients. Cluster 5 represents a smaller population of obese female patients with BMI mean (SD) 32.4 (3.2) kg∙m^-2^ with mildly impaired kidney function of eGFR 75.9 (25.3) ml∙min∙1.73m^-2^, while cluster 7 included the majority of female patients, who were older (mean (SD) 70.5 (8.9) years), had lower BMI of 24.9 (2.5) kg∙m^-2^) and lower kidney function with an eGFR of 59.7 (17.7) ml∙min∙1,73m^-2^. Conversely, all other clusters are exclusively (cluster 2) or predominantly male (clusters 1, 3, 4, 6, and 8). Clusters 1 and 6 are comprising subgroups with extremely poor preoperative kidney function and very high urea levels, including patients requiring dialysis. Cluster 2 represented a younger, male population with good kidney function (eGFR 102.8 (24.4) ml∙min∙1,73m^-2^, low urea (5.8 (1.4) mmol/l), and high haemoglobin (9.0 (0.7) mmol/l). This cluster contrasts with cluster 3, consisting of older male patients with poorer kidney function (age 66.0 (8.9) years, eGFR 75.4 (22.5) ml∙min∙1,73m^-2^), high inflammatory markers (leukocytes 10.6 (5.5) 10^3/l) and higher urea levels 7.3 (2.3) mmol/l. Groups 4 and 8 represent the oldest male group and a group with marked anaemia (Hb 5.6 (1.2) mmol/l), respectively.

**Table 3:** Patient characteristics and preoperative factors based on putative pre-cooling cluster membership for 4351 patients.

	Cluster 1	Cluster 2	Cluster 3	Cluster 4	Cluster 5	Cluster 6	Cluster 7	Cluster 8
***Number of patients***	54	1045	508	1323	358	16	639	402
*Gender (male*,*%)*	80	100.0	99	99	**0.0**	94	**0.0**	99
*BMI (kg∙m* ^*-2*^ *)*	**26.4 (3.1)**	28.9 (3.7)	**25.9 (3.2)**	26.3 (2.9)	**32.5 (3.2)**	28.2 (3.5)	24.9 (2.5)	26.7 (3.0)
*Age (years)*	**71.5 (8.9)**	**55.9 (6.7)**	**66.0 (8.9)**	71.4 (5.7)	67.5 (8.9)	62.2 (10.1)	**70.5 (8.9)**	66.1 (8.9)
*Platelets (x10^9/l)*	250 (64)	235 (50)	337 (90)	212 (43)	254 (73)	236 (64)	260 (82)	153 (48)
*eGFR*	**31.5 (19.3)**	**102.8 (24.4)**	75.4 (22.5)	70.1 (17.5)	75.9 (25.3)	**8.5 (1.8)**	**59.7 (17.7)**	76.3 (22.3)
*Leukocytes (x10^3/l)*	9.2 (3.3)	**7.8 (2.1)**	**10.6 (5.5)**	7.2 (1.7)	8.6 (2.8)	8.7 (1.8)	**8.2 (2.8)**	8.5 (3.4)
*Hb (mmol/l)*	7.4 (1.3)	**9.0 (0.7)**	8.2 (0.9)	**8.7 (0.8)**	7.8 (1.1)	6.9 (0.6)	**7.4 (1.3)**	**5.6 (1.2)**
*Urea (mmol/l)*	**24.8 (15)**	**5.8 (1.4)**	**7.3 (2.3)**	7.0 (2.0)	6.9 (2.7)	**24.6 (5.6)**	**7.4 (2.3)**	7.0 (2.4)
Creatinine (μmol/l)	265 (141)	**94 (17)**	102 (27)	102 (22)	91 (30)	972 (241)	**86 (23)**	**104 (31)**
*30-days mortality*	12.9^#$^	0.8^$^	4.1^#^	2.3	2.2	18.8^#$^	2.7	2.5
*5-year mortality*	64.8^#$^	5.2^$^	20.9^#^	16.2^#^	10.6^$^	50.0^#$^	15.8^#^	17.4^#^
***Cluster characterization***	Patients with moderate-to-severe decrease in kidney function	Overweight, younger male patients with good kidney function and low urea	Male patients with mild kidney function impairment, high inflammatory markers and urea	Older, male patients with moderate-normal lab values	Obese female patients with a mild kidney function impairment	Patients with kidney failure	Older female patients with a moderate decrease in kidney function	Male patients with marked anaemia

Results are presented as mean(SD), results that are distinct for a particular cluster are bolded for emphasis. Comparisons are based on analysis of variance or chi-squared tests. Pairwise-comparison between clusters for mortality are calculated with the Tukey HSD test. Clusters marked with # differed significantly compared to cluster 2, and clusters marked with $ differed significantly compared to cluster 3, all with p <0.001.

### 30-day and 5-year survival in pre-cooling phenotypes

**Fig 5:**
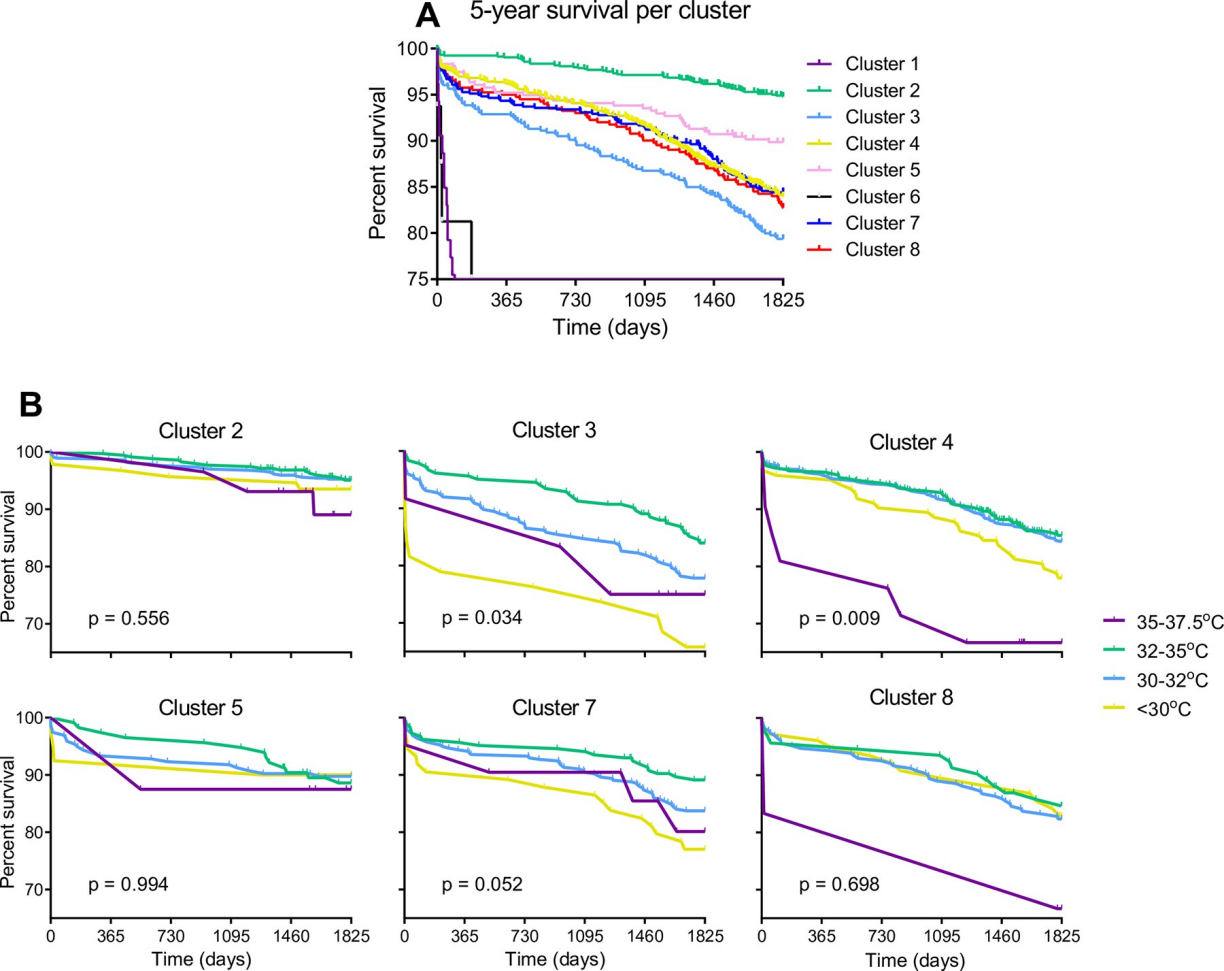
5-year survival per pre-cooling cluster. (a) 5-year survival is shown and differentiated for all 8 machine learning-based clusters. (b) survival for different nasopharyngeal temperature during CPB within each pre-cooling phenotype. Statistically tested using the long-rank test.

Amongst the 8 identified clusters, 4 different survival patterns were found (Fig 5A). Compared to the mean mortality of the entire cohort (30-days: 2.7%, 5-year: 14.4%), clusters 1 and 6, consisting of respectively 54 and 16 (pre)dialysis patients, showed the highest long-term mortality (64.8% and 50.0%, respectively). In addition, cluster 3, consisting of males with poor kidney function and increased inflammatory markers, had a significantly higher mortality (30-days: 4.1%; p <0.001, 5-year: 20.9%; p <0.001). In contrast, cluster 2 (younger males with the best kidney function and low inflammatory markers) had a significantly lower mortality (30-days: 0.8%; p <0.001, 5-year: 5.2%; p <0.001) compared to all other groups except cluster 5. Cluster 5 (all female, high BMI) had a significantly higher 5-year survival rate (10.6%; p <0.001) than cluster 3, an all-male cluster with similar pre-cooling parameters but lower BMI.

### Temperature strategies for different patient phenotypes

Next, we explored whether specific patient sub-groups might benefit from different CPB cooling strategies. Similar to the full cohort, mild hypothermia was significantly associated with increased survival in cluster 3 and 4 (Fig 5B, p = 0.034 and 0.009, respectively). Interestingly, the same was observed in all other clusters with mild hypothermia being associated with the highest survival, although not reaching significance due to a small number of patients. In addition, similar to the full cohort, both patients with the normothermic and severe hypothermic Tnp showed the lowest survival rates. However, although the highest survival is found at mild hypothermia in all patient clusters, the phenotypes with the highest 5-year mortality benefit to a larger extent from mild hypothermia during CPB (e.g. compare clusters with lowest mortality (2 and 5) with those having the highest (clusters 3 and 4)). Therefore, these data suggest that a nasopharyngeal temperature of 32°C during CPB is optimal, irrespective of the patient cluster profile.

## Discussion

This study documents the association of the lowest nasopharyngeal temperature in CPB assisted CABG on short (30-day) and long-term (5-year) postoperative survival. Our cohort study shows an optimal survival in patients subjected to mild hypothermia (32–35°C) during CPB. Further analysis of mortality rate per degree Celsius showed that survival was optimal in patients perfused at 32–33°C and in patients with intermediate cooling rates. Additionally, cluster analysis identified eight patient subgroups with different risk profiles, all showing optimal survival at mild hypothermia during CPB. These outcomes indicate a clear influence of intraoperative temperature on outcome, which merits further exploration in a randomized controlled trial.

Although cooling is widely used in cardiac operations to prevent ischemic damage, optimal temperature treatment is still debated [16]. A previous study [17] and systematic review [18] found no impact of CPB cooling on 30 day mortality risk compared to normothermia during CPB. However, patient groups in these studies covered large, binary temperature ranges of 33–37°C *vs*. 25–30°C and > 34°C *vs*. < 34°C, respectively. Because of the large dataset and long-term follow-up, we were able to compare outcomes of a multiple CPB temperature categories, which entails a more detailed approach than previous datasets. Given the excess mortality in the lowest and highest temperature categories in our study, this may explain the observed absence of mortality risk differences in earlier studies.

So far, the mechanism conferring the beneficial effects of mild hypothermia are unresolved. Previous research has documented immediate post-operative effects [19–22] and showed mild hypothermia during CABG procedures to shorten intensive care stay [19] and improve the neurological outcome [20], when compared with normothermia. Contrary, low CPB temperatures (≤28°C) were associated with increased risk of stroke [23] and AKI [23]. In addition, temperature >37°C was also associated with AKI [7]. Yet, identification of underlying factors is complex, as exemplified by lower levels of systemic inflammatory mediators [21], including plasma IL-6 and IL-8 cytokines and soluble adhesion molecules, in mild hypothermia and normothermia, compared to severe hypothermia [22]. Furthermore, ischemia/reperfusion and reactive oxygen species formation are suggested as important causative factors [3, 24].

Interestingly, we observed a large variation in cooling and rewarming rates of patients, irrespective of the lowest body temperature reached during CPB. Various underlying factors may be involved, such as BMI, peripheral circulation and autonomic regulation, possibly reflecting the initial general health status of the patient. In our cohort, both the slowest and fastest cooling rates were associated with increased mortality, whereas no significant different survival was found between different rewarming rates. Clearly, further research is required to understand the significance of the observed differences and whether these can be exploited to optimize outcome.

Machine learning based clustering analysis identified clusters with either female or male patients, and thus contributes to the increasing awareness of gender-related differences. In both genders, mortality rate of subgroups increased with advanced age, lower kidney function, extreme BMIs at both ends of its range and higher inflammatory markers. The subgroup with lowest mortality identified for both genders consisted of younger, slightly overweight patients with good or mildly impaired kidney function. Importantly, all subgroups displayed a similar association between CPB temperature and survival as the overall group, suggesting that its beneficial effect is independent of gender. A better understanding of risk profiles by gender may be important for optimal perioperative management of both male and female CABG patients [25].

### Limitations

We only included routinely available patient characteristics and peri-operative data, which enabled us to build a uniform and reliable database. Co-morbidity and medication data were unreliable in completeness and collection and hence not included, although they are partly reflected in the included EuroScore. Likewise, data on detailed cardiac performance such as ejection fraction was not included. We cannot infer their influence on the association between body temperature and mortality. The large dataset that we used may have well averaged out their contribution to outcome in all temperature categories.

Recruitment of patients from a single centre may limit the generalizability of the current study. Despite the identification of temperature as an independent predictor of survival, this only indicates association and not causality. Further, all patients were operated with CPB, which limits generalizability our findings to other forms of CABG operations, such as off-pump procedures.

Unfortunately, patient specific details of the surgical team regarding temperature management are not known in our cohort and may have potentially influenced the outcomes. Yet, as shown in Table 1, no clinically relevant preoperative differences or EuroSCORE were observed between the 4 temperature categories. Additionally, our cluster analysis based on pre-surgery patient factors demonstrated improved survival of mild hypothermia in all clusters. Together, these data substantiate an absence of patient related preferences for a particular temperature strategy.

Collectively, our study demonstrates the need for prospective RCTs to optimize the impact of cooling during CPB on survival after cardiac operations.

## Conclusion

In conclusion, we showed that intraoperative mild hypothermia is associated with improved 30-days and 5 year survival following CPB-assisted CABG surgery. Nasopharyngeal CPB temperatures of 32 and 33°C were associated with the highest survival rates, independently of patient phenotype and gender. Moreover, Machine Learning based clustering analysis on routinely collected pre-cooling data clearly differentiated mortality risk of particular patient groups and identified gender as a clear differentiating factor. We suggest a RCT to confirm the optimal CPB temperature.

## Supporting information

S1 Fig Visualization of the putative clusters using principal component analysis. For each value of k between 2 and 9, a different number of clusters was generated. This visual representation was one of the elements used in determining the optimal number of clusters.
(TIFF)

S2 Fig Heatmap showing the variables characterizing each cluster. Values are normalized and scaled between -3 and 2. Light blue coloring represents lower values, and purple coloring represents higher values.
(TIFF)

S3 Fig Density plot showing aortic cross-clamp time (min) related to temperature category and survival for 31 days mortality and 5 year (1825 days) mortality. (PDF)

S1 File Containing additional descriptive information about K-mean clustering and S1 Table, multivariate COX regression analysis, including year of surgery. (DOCX)
