# Supplementary figures and images for: Mild hypothermia during cardiopulmonary bypass assisted CABG is associated with improved short- and long-term survival, a 18-year cohort study
Source: PLoS One. 2022 Aug 25;17(8):e0273370. doi: 10.1371/journal.pone.0273370 (PMC9409584; doi:10.1371/journal.pone.0273370)

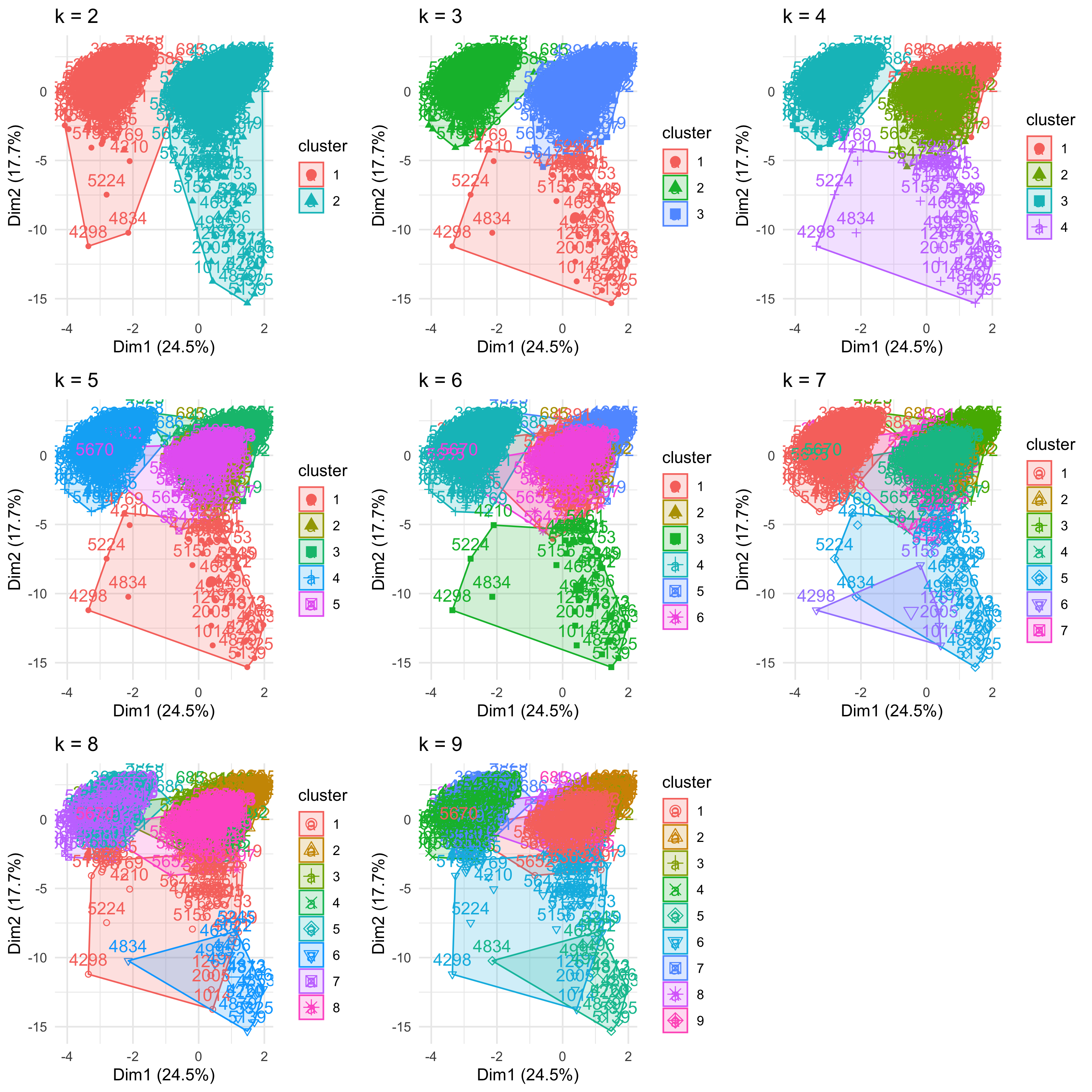

Supplement: S1 Fig — For each value of k between 2 and 9, a different number of clusters was generated. This visual representation was one of the elements used in determining the optimal number of clusters. (TIFF) [file pone.0273370.s001.tiff]

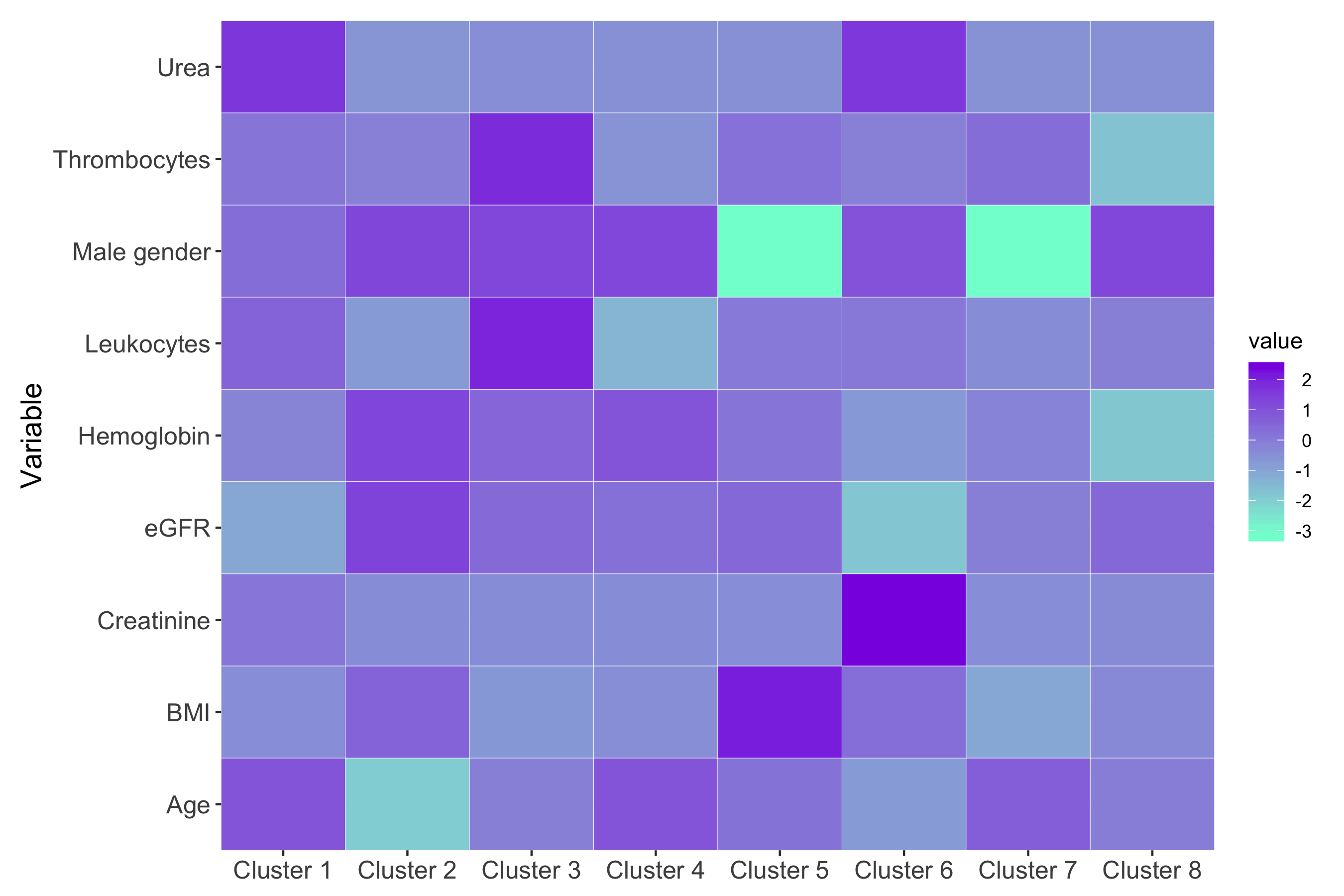

Supplement: S2 Fig — Values are normalized and scaled between -3 and 2. Light blue coloring represents lower values, and purple coloring represents higher values. (TIFF) [file pone.0273370.s002.tiff]

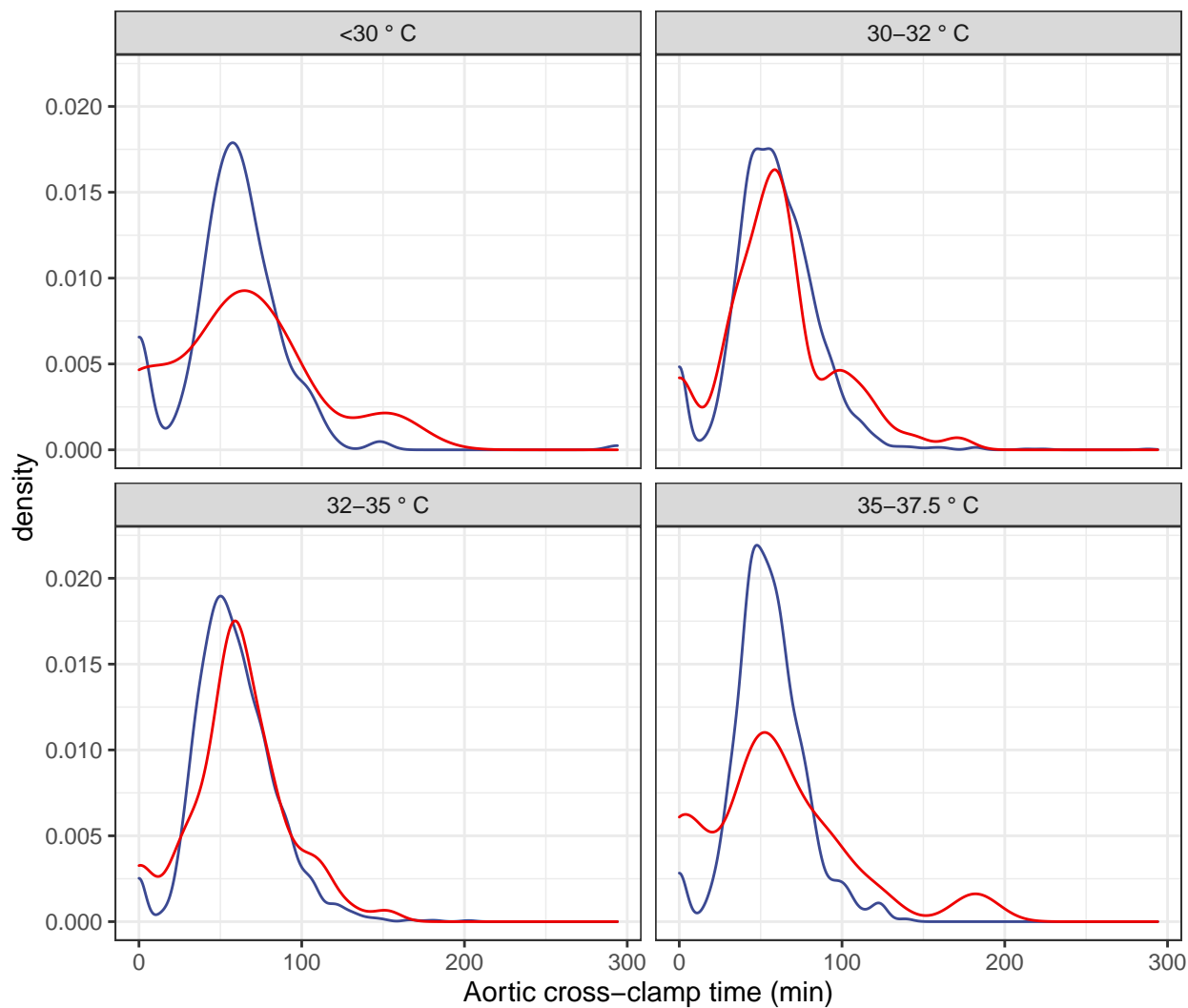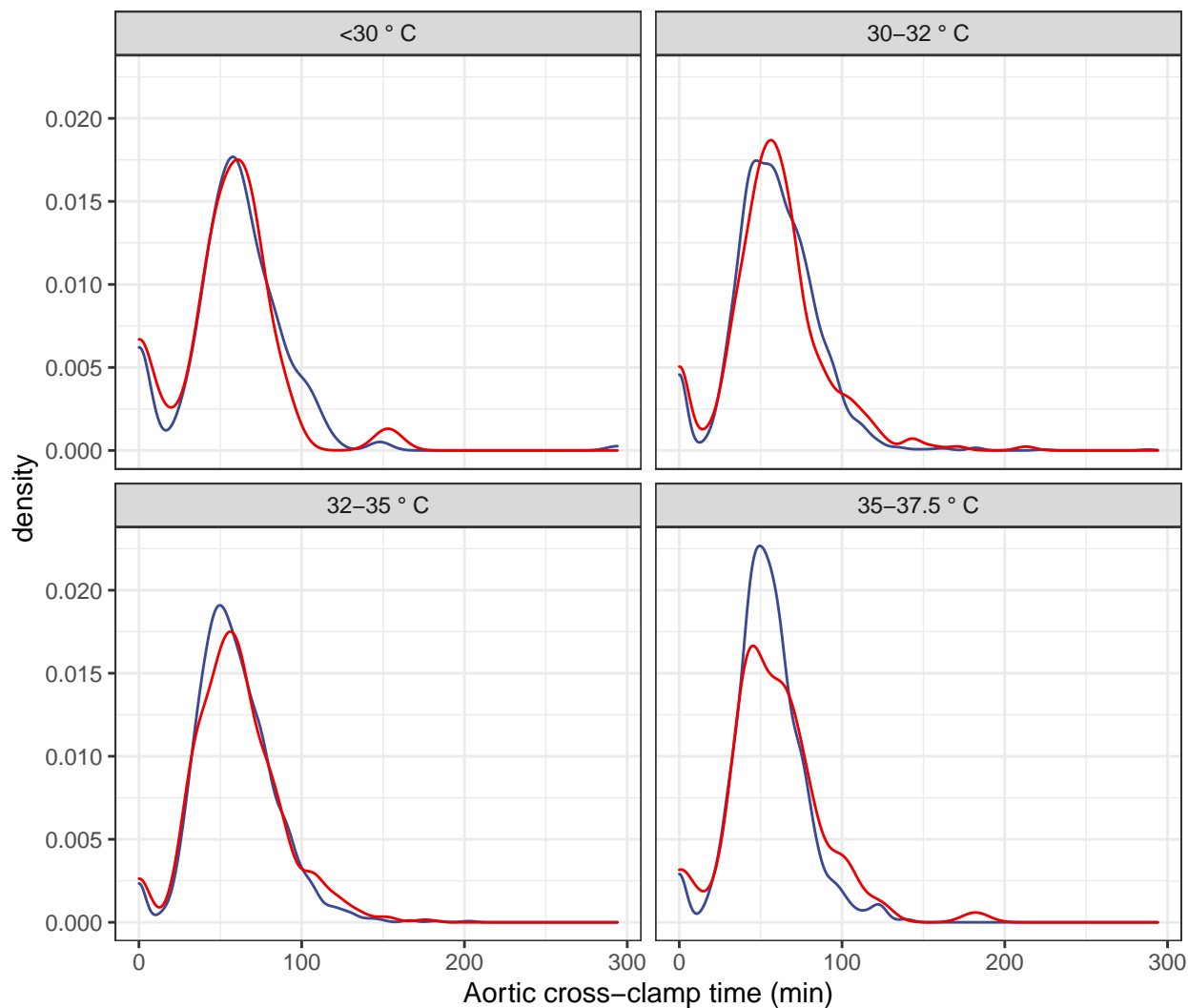

Supplement: S3 Fig — (PDF) [file pone.0273370.s003.pdf]
