# Supplementary material for: Mild hypothermia during cardiopulmonary bypass assisted CABG is associated with improved short- and long-term survival, a 18-year cohort study
Source: PLoS One. 2022 Aug 25;17(8):e0273370. doi: 10.1371/journal.pone.0273370 (PMC9409584; doi:10.1371/journal.pone.0273370)
Supplement: S1 File — (DOCX) [file pone.0273370.s004.docx]

**Supplemental Material***Mild hypothermia during cardiopulmonary bypass assisted CABG is associated with improved short- and long-term survival, a 18-year cohort study*

**Additional information about K-mean clustering**

Clustering analysis is a form of unsupervised machine learning that has been applied extensively to explore and characterize patient phenotypes in multiple medical field, from including cancer to critical care and asthma. Clustering algorithms are a group of multivariate mathematical algorithms that seek to quantify the similarity between datapoints (patients within a cohort) based on multiple input variables. As a data-driven approach, it generates novel subgroups without any a priori hypotheses. In other words, clustering methods group data in a way that may be useful, without the user specifying how this grouping should occur. There are two categories of clustering algorithms (“hard” and “soft”), with the difference being how they divide the data. In hard clustering algorithms such as k-means, each data point can belong to one cluster only, whereas soft clustering algorithms assign data points a membership probability of belonging to one or more clusters. After clusters have been defined by the algorithm, internal validity has to be tested. Internal validity is assessed by verifying whether the structure of the clustering is intrinsically appropriate for the data. In other words, by checking if data points are, simultaneously, similar within the same cluster while being as distinct as possible from those in other clusters. This can be measured through indices such as the Silhouette and Dunn indices, which will define what the ideal number of clusters for the data is.

**Supplementary Table 1. Multivariate COX regression analysis, including year of surgery**

|  | **30-days survival** HR (95% CI) | **p-value** | **5-year survival** HR (95% CI) | **p-value** |
| --- | --- | --- | --- | --- |
| **Patient characteristics** |  |  |  |  |
| Age (years) | 1.07 (1.04-1.09) | <0.001 | 1.06(1.05-1.07) | <0.001 |
| Female gender | 0.84 (0.56-1.28) | 0.424 | 0.71 (0.59-0.85) | 0.018 |
| BMI (kg∙m^-2^) | 0.98 (0.93-1.03) | 0.372 | 0.99 (0.98-1.02) | 0.70 |
|  |  |  |  |  |
| **Preoperative parameters** |  |  |  |  |
| Hemoglobin (mmol/l) | 1.01 (0.89-1.16) | 0.837 | 0.91 (0.86-0.96) | 0.001 |
| Platelet count (x10^9/l) | 1.00 (1.00 -1.01) | 0.449 | 1.00 (1.00-1.01) | <0.001 |
|  |  |  |  |  |
| **Acute kidney injury** | 4.93 (3.40-7.16) | <0.001 | 2.29 (1.91-2.75) | <0.001 |
|  |  |  |  |  |
| **Perioperative parameters** |  |  |  |  |
| Duration of perfusion   (15 min) | 1.16 (1.11-1.20) | <0.001 | 1.10 (1.08-1.13) | <0.001 |
|  |  |  |  |  |
| **Surgery year** |  |  |  |  |
| 1997-2000  2001-2004  2004-2008  2008-2012  2012-2015 | Reference  0.80 (0.49-1.29)  0.54 (0.3-0.96)  0.65 (0.34-1.27)  0.43 (0.12-1.50) | -  0.359  0.035  0.211  0.185 | Reference  0.90 (0.73-1.11)  0.72 (0.57-0.91)  0.96 (0.72-1.26)  0.37 (0.136-1.03) | -  0.33  0.005  0.744  0.057 |
|  |  |  |  |  |
| **Temperature strategy** |  |  |  |  |
| Normothermia (35-37°C)  Mild hypothermia (32-35°C)  Moderate hypothermia (30-32°C)  Severe hypothermia (<30°C) | Reference  0.17 (0.08-0.34)  0.20 (0.10-0.40)  0.40 (0.18-0.85) | -  <0.001  <0.001  0.018 | Reference  0.37 (0.26-0.52)  0.42 (0.30-0.60)  0.51 (0.34-0.76) | -  <0.001  <0.001  0.001 |
|  |  |  |  |  |

Hazard ratio (HR) with 95% confidence interval (CI) on mortality in CABG surgery.
